# Impact on Bacterial Community in Midguts of the Asian Corn Borer Larvae by Transgenic *Trichoderma* Strain Overexpressing a Heterologous *chit42* Gene with Chitin-Binding Domain

**DOI:** 10.1371/journal.pone.0055555

**Published:** 2013-02-15

**Authors:** Yingying Li, Kehe Fu, Shigang Gao, Qiong Wu, Lili Fan, Yaqian Li, Jie Chen

**Affiliations:** 1 Department of Resource and Environmental Science, School of Agriculture and Biology, Shanghai Jiaotong University, Shanghai, China; 2 Key Laboratory of Urban Agriculture (South), Ministry of Agriculture, China; U. Kentucky, United States of America

## Abstract

This paper is the first report of the impact on the bacterial community in the midgut of the Asian corn borer (*Ostrinia furnacalis*) by the chitinase from the transgenic *Trichoderma* strain. In this study, we detected a change of the bacterial community in the midgut of the fourth instar larvae by using a culture-independent method. Results suggested that Proteobacteria and Firmicutes were the most highly represented phyla, being present in all the midgut bacterial communities. The observed species richness was simple, ranging from four to five of all the 16S rRNA clone libraries. When using *Trichoderma* fermentation liquids as additives, the percentages of the dominant flora in the total bacterial community in larval midgut changed significantly. The community of the genus *Ochrobactrum* in the midgut decreased significantly when the larvae were fed with the fermentation liquids of the transgenic *Trichoderma* strain Mc4. However, the *Enterococcus* community increased and then occupied the vacated niche of the *Ochrobactrum* members. Furthermore, the Shannon–Wiener (H) and the Simpson (1-D) indexes of the larval midgut bacterial library treated by feeding fermentation liquids of the transgenic *Trichoderma* strain Mc4 was the lowest compared with the culture medium, fermentation liquids of the wild type strain T30, and the sterile artificial diet. The *Enterococcus* sp. strain was isolated and characterized from the healthy larvae midgut of the Asian corn borer. An infection study of the Asian corn borer larvae using *Enterococcus* sp. ACB-1 revealed that a correlation existed between the increased *Enterococcus* community in the larval midgut and larval mortality. These results demonstrated that the transgenic *Trichoderma* strain could affect the composition of the midgut bacterial community. The change of the midgut bacterial community might be viewed as one of the factors resulting in the increased mortality of the Asian corn borer larvae.

## Introduction

The Asian corn borer [*Ostrinia furnacalis* (Guenée)] is a Lepidopteran insect distributed in East and Southeast Asian countries, including countries such as China, Japan, Korea, Thailand, the Philippines, Indonesia, Malaysia, as well as some islands in the Pacific Ocean [1]. The species remains the most significant economic insect pest for corn. Our previous study revealed that an overexpression of the *Metarhizium anisopliae chit42* gene with a chitin-binding domain (chBD) in *Trichoderma koningii* increases the biocontrol activity, particularly the virulence against the Asian corn borer larvae [2]. Transgenic *Trichoderma* could also hinder the growth and development of the larvae. We speculated that the bacterial community of the alimentary tract might be one of the important factors for insect health. Studies on bacterial communities focusing on the Lepidopteran midgut are limited [3], [4], [5], [6]. To date, no reports are available on the change of the bacterial diversity in the midgut of the Asian corn borer larvae induced by the transgenic *Trichoderma* strain overexpressing entomopathogenic genes.

Insect digestive system is a complex ecosystem harboring a large number of microorganisms. These microorganisms constitute communities. Microflora in the digestive system is an important factor in the nutritional, immune, and defense functions under normal circumstances [7], [8]. On one hand, the nutritional physiology of the insects is closely linked with their gut microorganisms [9]. The community structures and metabolic activities of microorganisms are affected by the gut microenvironment of the insect. On the other hand, intestinal microorganisms can also affect the physiological activity of the insect. Studies on the microbial diversity of the insect midgut are limited and primarily focus on termites [10], aphids [11] and honeybees [12].

Microbial communities are dynamic. Feeding, digestion, and excretion can facilitate changes in composition and structure [4]. These changes can be attributed to a variety of factors, including nutrient availability, physical aspects of the environment, and proximity to other organisms [13], [14], [15]. Chitinases from *Trichoderma* and *Metarhizium* have been proven useful in biocontrol activities against a wide range of plant fungal pathogens [16] and insects [17]. Therefore, chitinase from both sources seemed to be an adverse factor facilitating changes of the gut bacterial composition and structure.

Our previous study revealed that an overexpression of the *Metarhizium anisopliae chit42* gene with a chitin-binding domain in *Trichoderma koningii* displayed an increased biocontrol activity, particularly in the virulence against the Asian corn borer larvae. However, little is known about the anti-insect mechanism of the transgenic *Trichoderma* strain in terms of a midgut microbial ecology. In this study, we used a culture-independent method to detect the change in whole bacterial community of the fourth instar Asian corn borer larvae in response to the fermentation liquid from the transgenic *Trichoderma* strain overexpressing the *chit42* gene with a chBD.

## Materials and Methods

### Microbial Strains

The *Escherichia coli* host strain DH5 α was grown in an LB medium. Wild type (WT) *T. koningii* T30 was provided by the Biocontrol Laboratory of Plant Diseases, Shenyang Agricultural University, China. This species has been used for the biocontrol of soil-borne diseases in a variety of vegetable crops. The *chit42* gene was cloned from *M. anisopliae*. The chBD from bitter melons (*Momordica charantia*) was fused with the *chit42* gene in the C-terminal to constitute the hybrid chitinase gene. The hybrid chitinase gene was controlled under the TrpC promoter and TrpC terminator to constitute an overexpression cassette. The overexpression cassette was transformed into the WT *Trichoderma* strain *T. koningii* T30 by using an Agrobacterium-mediated transformation technique. The *Trichoderma* transformants harboring the hybrid chitinase gene overexpression cassette were selected and labeled as *T. koningii* Mc4. *T. koningii* Mc4 had been proven to have an enhanced insecticidal activity against the Asian corn borer larvae and antifungal capability toward *Fusarium verticillioides* and *Rhizoctonia solani* [2].

### Preparation of Fermentation Liquids from *Trichoderma* Strain

Mycelia of the WT strain *Trichoderma* T30 and transgenic *Trichoderma* strain Mc4 each were isolated and transferred into a 100 mL fermentation medium (MgSO_4_.7H_2_O 0.6 g/L, FeSO_4_.7H_2_O 0.1 g/L_,_ NH_4_NO_3_ 3.0 g/L, KH_2_PO_4_ 2.0 g/L, colloidal chitin 10% v/v, powdered chitin 2% w/w), inoculated at 28°C for 180 rpm in a shaking table at constant temperature until the highest chitinase activity was achieved. The highest chitinase activity values of T30 and Mc4 were 12.4 U/mL and 46.8 U/mL, respectively. One unit of chitinase activity is defined as the amount of enzyme required to release 1 µmol of *N*-acetyl-ß-glucosamine per hour. Fermentation liquids were prepared by collecting the supernatant after removing insoluble substances as well as the debris of the mycelium by centrifugation at 4°C, 12000 g for 30 min. The artificial diets were mixed with the fermentation liquids of T30 and Mc4 (artificial diet/fermentation liquids = 1∶1.2 w/v), whereas the artificial diets mixed with the supernatant of the control fermentation medium was used as the control. The Asian corn borer larvae reared on the artificial diets without any additives were used as the control.

Chitinase produced by *Trichoderma* in the fermentation medium could degrade chitin into *N*-acetyl-D-(+)-glucosamine (NAG). This derivative could more easily be used as a carbon source compared with chitin. A 3, 5-dinitrosalicylic acid method was used to determine the NAG content in the fermentation liquids. The chitinase fermentation liquids were treated by boiling for 5 min to inactivate the enzyme content. The effect of enzyme-inactivated fermentation liquids on the midgut bacterial community of fourth instar larvae was analyzed against the control treatment, in which larvae were reared on the sterile artificial diets.

### Insect Rearing and Treatments

Second instar larvae of the Asian corn borer were obtained from the Institute of Plant Protection, Chinese Academy of Agricultural Sciences, and were reared on the sterile artificial diet with for 14 h: 10 h (light: dark) at 25°C under 40% to 60% relative humidity until the fourth instar.

Strong and healthy larvae with the similar sizes were selected and starved for 24 h before being fed with the artificial diets under different treatments. The selected larvae were divided into six groups. Each group contained 20 larvae, among which 10 were kept for further feeding on sterile artificial diets. Another 10 were selected for comparison of the midgut bacterial libraries between different treatments. The treatments included larvae reared on the sterile artificial diets as well as artificial diets supplemented with the supernatant of the control fermentation liquids, artificial diets supplemented with the supernatant of the fermentation liquids of *Trichoderma* WT strain T30, artificial diets supplemented with the supernatant of the fermentation liquids of the transgenic *Trichoderma* strain Mc4, artificial diets supplemented with the supernatant of the enzyme-inactivated fermentation liquids of T30, and artificial diets supplemented with the supernatant of the enzyme-inactivated fermentation liquids of Mc4. All feeding treatments were completed for up to three days. The experiments were repeated thrice.

### Midgut Separation and DNA Extraction

Fourth instar larvae were placed in sterilized culture dishes and starved for 6 h to reduce the artificial diets content. The larvae were surface-sterilized and dissected with anatomic scissors and forceps. The midguts were removed and stored at −80°C prior to DNA isolation. Total microbial DNA was extracted from the midgut by using the protocol mentioned in Broderick et al. [3]. DNA was quantified and adjusted to 10 ng/µL [18] using Quant-iT™ PicoGreen® dsDNA Reagent and Kits (Invitrogen). A 2× PicoGreen working reagent was prepared by adding 5 µL of a 200-fold dilution into 995 µL of 1× TE buffer. Thereafter, 10 µL of the DNA extracts were added into 990 µL of 1× TE buffer to constitute the DNA sample. Subsequently, 1 mL of the DNA sample was added into 1 mL of the 2× PicoGreen working reagent and reacted for 2 min at room temperature and protected from light. The sample fluorescence was measured using a spectrofluorometer. The DNA standard curve was prepared using a lambda DNA.

### Construction of the Midgut16S rRNA Gene Library

The midgut 16S rRNA gene libraries of the fourth instar larvae after treatment were constructed. Total DNA of the samples was separately PCR-amplified by using the primers 27F and 1492R [19]. PCR reactions were performed in a 25 µL solution containing 1 ng/µL DNA, 1× PCR reaction buffer (TaKaRa, Japan), 0.5 mM additional MgCl_2_ (TaKaRa, Japan), 0.2 µM of each primer (Sangon Biotech, Shanghai, China), 0.4 mM of each dNTP (TaKaRa, Japan), and 1 U of rTaq DNA polymerase (TaKaRa, Japan). PCR was performed in vapor (Eppendorf, Germany) under appropriate conditions: starting at 94°C for 5 min, followed by 30 cycles at 94°C for 30 s, 55°C for 30 s, 72°C for 1 min and 30 s, and 72°C for 10 min. The purified PCR products were cloned into a pMD19-T simple vector system (TaKaRa, Japan) according to the manufacturer’s instructions. The ligation products were transformed into the competent *E. coli* DH5α cells. The clones were screened using an LB medium containing ampicillin (100 µg/L) with isopropyl-β-D-thiogalactopyranoside and 5-bromo-4-chloro-3-indolyl-β-D-galactopyranoside. At least 150 white clones were selected randomly for each clone library.

### Phylogenetic Analysis of the Midgut16S rDNA Gene Library

The PCR products amplified from each clone were digested by two Fast-Digest restriction enzymes (*Hin*fI and *Afa*I, 37°C for 30 min). The digestion reactions (30 µL of the total volume) contained 15 µL of the PCR products, 3 µL of 10× Fast-Digest buffer (Fermentas), 1 µL of the Fast-Digest enzyme (*Hin*fI or *Afa*I, Fermentas), and 11 µL of dH_2_O. The restriction enzyme analysis of the positive clones in the 16S rDNA library was conducted through an agarose gel electrophoresis (2%). All the positive clones were classified according to the patterns of the restriction fragments digested with the restriction enzymes in agarose gel. The clones that exhibited the same patterns of restriction fragments after digestion were classified into the same operational taxonomic units (OTUs). The sequences were initially analyzed using SeqMan and then rapidly aligned using the Greengenes Web application (http://greengenes.lbl.gov). Chimeras were verified against Ribosomal Database Project II and then removed from the group for further analysis.

An estimate of the library was employed to assess whether the constructed midgut 16S rDNA gene libraries were sufficiently large by using the formula
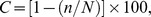
where *C* is the percent coverage, *n* is the number of sequence appearing once in each library, and *N* is the total number of clones in each library [20].

S_Chao1_ was calculated for each clone library to assess whether the libraries were sufficiently large to yield a stable estimate of the phylotype richness using a Web-based program (http://www.aslo.org/lomethods/free/2004/0114a.html). PAST software was used to calculate the Shannon–Wiener (H) and the Simpson (1-D) indexes for each of the libraries constructed [21].

The sequences from each OTU were submitted to GenBank for BLAST analyses [22]. Most of the associated sequences were downloaded in a FASTA format and then aligned using the program MEGA 4.0. The aligned tests were converted to the MEGA format before the phylogenetic trees were constructed using the neighbor-joining method with 1000 bootstrap replicates [23].

### Analysis of the Microflora Distribution

All the positive clones in the library were PCR-amplified using the universal primers in the pMD19-T simple vector. The PCR products were digested. Clones belonging to the same OTU were counted. The percent of microflora distribution was obtained using the formula
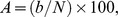
where *A* is the percentage of microflora distribution in each clone library, *b* is the total clone numbers belonging to the same OTU in each library, and *N* is the total number of clones in each library.

### Bacterial Isolation from the Midgut of the Asian Corn Borer Larvae

*Enterococcus* sp. was isolated from the midgut of the healthy Asian corn borer larvae. The cutting instruments, including anatomic scissors and forceps, were disinfected by immersion in 95% ethanol. Larvae surface were sterilized before dissecting the midguts using anatomic scissors and forceps. The digestive tract was shredded and deposited in an Eppendorf tube containing 200 µL of sterile saline solution. The decimally diluted digestive tract solutions were mixed with the *Enterococcus* Agar (Qingdao Hope Biol-Technology Co., Ltd, China) to isolate and culture the *Enterococcus* sp. from different species. The plates were sealed with parafilm and then incubated at 37°C for at least 24 h.

### Phylogenic Analysis of *Enterococcus* sp. Isolated from the Larval Midgut

*Enterococcus* sp. was cultured in the *Enterococcus* broth. The genomic DNA of *Enterococcus* sp. was extracted according to the protocols by using a Wizard® Genomic DNA Purification Kit (Promega, USA). The 16S rRNA gene sequence of *Enterococcus* sp. was PCR-amplified using the primers 27F and 1492R. PCR reactions were performed in a system volume of 25 µL system according to the standard protocols under approximate conditions. The PCR products were purified and then ligated with the pMD19T simple vector. Positive clones were obtained by transforming the ligation products into *E. coli* DH 5α competent cells. The 16S rRNA sequences of *Enterococcus* sp. was submitted to GenBank for BLAST analyses. Most associated sequences were downloaded in a FASTA format and then aligned using the program MEGA 4.0. The aligned tests were converted to the MEGA format before phylogenetic trees were constructed using the neighbor-joining method with 1000 bootstrap replicates.

### Infection of the Asian Corn Borer Larvae Using *Enterococcus* sp. Under Germfree Conditions

The Asian corn borer larvae were reared on the artificial diets until fourth instar. Larvae with similar dimensions and sizes were selected and starved for 24 h before conducting the infection study. A disinfection test on the body surface of the larvae was performed. The infection study with the isolated *Enterococcus* sp. was performed on the healthy larvae, which were kept at 25°C and then fed with the sterile artificial diets under germfree conditions. Dilutions (10^−3^, 10^−5^, and 10^−7^) of an overnight pure culture of *Enterococcus* sp. were harvested and suspended in physiological saline. These dilutions of *Enterococcus* sp. were mixed thoroughly with the sterile artificial diets. For each experiment, 20 larvae were used. All the experiments were conducted in triplicates with three biological repetitions. The control diets were supplemented with physiological saline and sterile distilled water.

The infectivity of the isolated *Enterococcus* sp. (dilution of 10^−5^) was also examined by injecting into the body cavity of the Asian corn borer larvae. A tuberculin syringe with fine intradermal needle was used to inject the body cavity of the larvae with 0.02 mL of the *Enterococcus* sp. suspension according to the methods described by Lysenko. The control diets were supplemented with physiological saline and sterile distilled water. For each experiment, 20 larvae were used. The experiments were repeated thrice.

### Nucleotide Sequence Accession Number

The 16S rRNA gene sequences from this study were deposited in GenBank under accession numbers JX679623 to JX679640. The 16S rRNA gene sequence of the isolated *Enterococcus* sp. in this study was deposited in GenBank under accession number KC243406.

## Results

### Construction of the Clone Libraries

**Figure 1:**
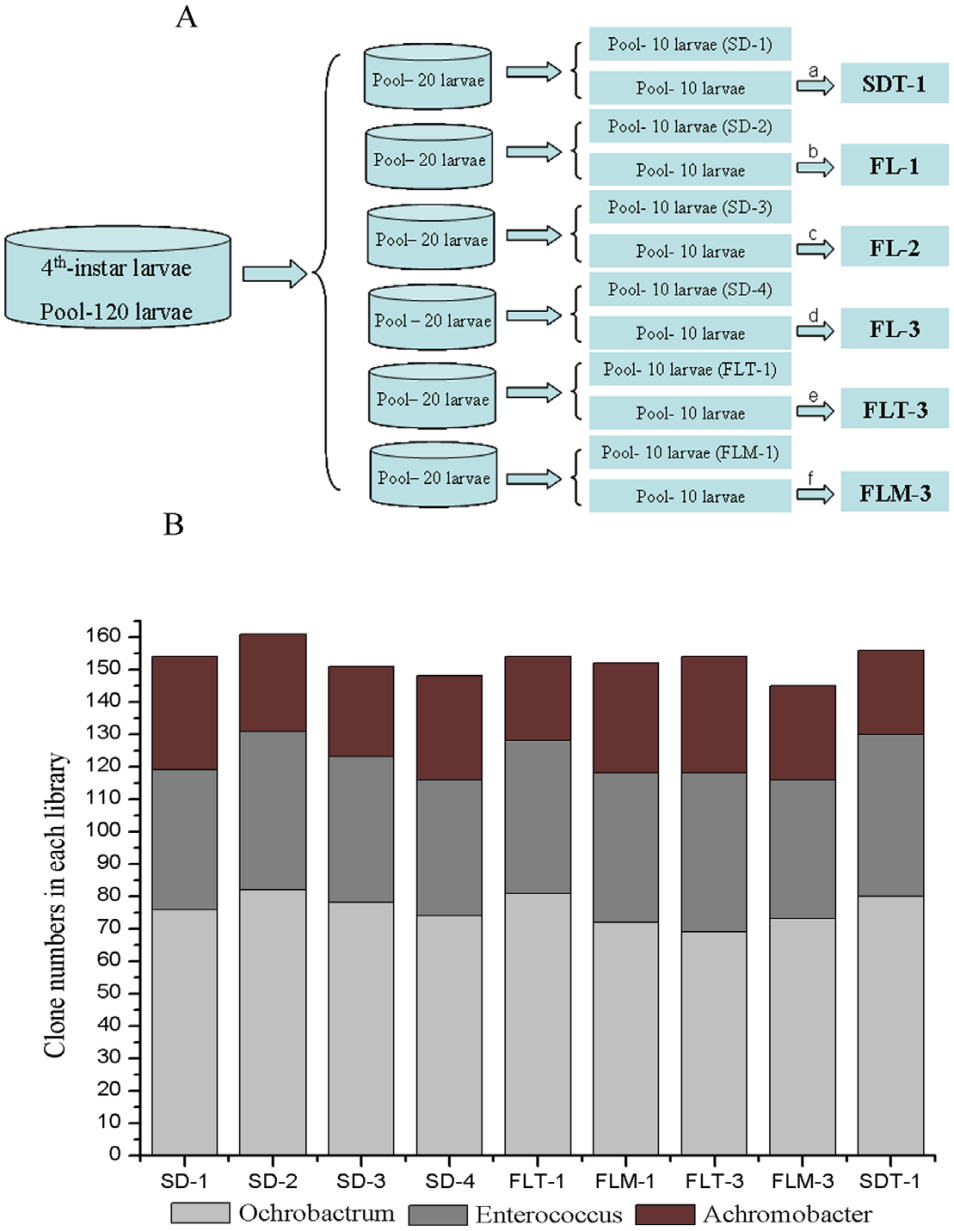
Construction and analysis of bacterial 16S rRNA clone libraries of larval midgut. (**A**) Construction strategy of larval midgut bacterial libraries. SD-1 (midgut bacterial library of fourth instar larvae reared on the sterile artificial diets in the first day of feeding), SD-2 (midgut bacterial library of fourth instar larvae reared on the sterile artificial diets in the first day of feeding), SD-3 (midgut bacterial library of fourth instar larvae reared on the sterile artificial diets in the first day of feeding), SD-4 (midgut bacterial library of fourth instar larvae reared on the sterile artificial diets in the first day of feeding), FLT-1 (midgut bacterial library of fourth instar larvae reared on the sterile artificial diets in the first day of feeding), and FLM-1 (midgut bacterial library of fourth instar larvae reared on the sterile artificial diets in the first day of feeding), SDT-1 (midgut bacterial library of fourth instar larvae reared on the sterile artificial diets for 3 d), FL-1 (midgut bacterial library of fourth instar larvae reared on the sterile artificial diets supplemented with the supernatant of the control fermentation liquids for 3 d), FL-2 (midgut bacterial library of fourth instar larvae reared on the sterile artificial diets supplemented with the supernatant of T30 fermentation liquids for 3 d), FL-3 (midgut bacterial library of fourth instar larvae reared on the sterile artificial diets supplemented with the supernatant of Mc4 fermentation liquids for 3 d), FLT-3 (midgut bacterial library of fourth instar larvae reared on the sterile artificial diets supplemented with the supernatant of the enzyme-inactivated T30 fermentation liquids for 3 d), and FLM-3 (midgut bacterial library of fourth instar larvae reared on the sterile artificial diets supplemented with the supernatant of the enzyme-inactivated Mc4 fermentation liquids for 3 d); a, fourth instar larvae reared on sterile artificial diets for 3 d; b, fourth instar larvae reared on sterile artificial diets supplemented with supernatant of control fermentation liquids for 3 d; c, fourth instar larvae reared on sterile artificial diets supplemented with supernatant of T30 fermentation liquids for 3 d; d, fourth instar larvae reared on sterile artificial diets supplemented with supernatant of Mc4 fermentation liquids for 3 d; e, fourth instar larvae reared on sterile artificial diets supplemented with supernatant of enzyme-inactivated T30 fermentation liquids for 3 d; f, fourth instar larvae reared on sterile artificial diets supplemented with supernatant of enzyme-inactivated Mc4 fermentation liquids for 3 d. (**B**) Comparison of dominant flora in the larval midgut between different clone libraries.

A total of 12 midgut bacterial libraries were obtained (Figure 1A) and called: SD-1 (midgut bacterial library of fourth instar larvae reared on the sterile artificial diets in the first day of feeding), SD-2 (midgut bacterial library of fourth instar larvae reared on the sterile artificial diets in the first day of feeding), SD-3 (midgut bacterial library of fourth instar larvae reared on the sterile artificial diets in the first day of feeding), SD-4 (midgut bacterial library of fourth instar larvae reared on the sterile artificial diets in the first day of feeding), FLT-1 (midgut bacterial library of fourth instar larvae reared on the sterile artificial diets in the first day of feeding), FLM-1 (midgut bacterial library of fourth instar larvae reared on the sterile artificial diets in the first day of feeding), SDT-1 (midgut bacterial library of fourth instar larvae reared on the sterile artificial diets for 3 d), FL-1 (midgut bacterial library of fourth instar larvae reared on the sterile artificial diets supplemented with the supernatant of the control fermentation liquids for 3 d), FL-2 (midgut bacterial library of fourth instar larvae reared on the sterile artificial diets supplemented with the supernatant of T30 fermentation liquids for 3 d), FL-3 (midgut bacterial library of fourth instar larvae reared on the sterile artificial diets supplemented with the supernatant of Mc4 fermentation liquids for 3 d), FLT-3 (midgut bacterial library of fourth instar larvae reared on the sterile artificial diets supplemented with the supernatant of the enzyme-inactivated T30 fermentation liquids for 3 d), and FLM-3 (midgut bacterial library of fourth instar larvae reared on the sterile artificial diets supplemented with the supernatant of the enzyme-inactivated Mc4 fermentation liquids for 3 d).

The bacterial communities were similar regardless of the larvae were collected from the first day of feeding on the sterile artificial diets (clone libraries of SD-1, SD-2, SD-3, SD-4, FLT-B-1, and FLM-B-1) or after feeding for 3 d on the same diets (clone library of SDT-1), or feeding on the artificial diets supplemented with fermentation medium without inoculation (clone library of FL-1). No obvious differences were observed when larvae were fed with the sterile artificial diets supplemented with the supernatant of enzyme-inactivated fermentation liquids for 3 d (clone library of FLT-B-3 and FLM-B-3), as compared with the larvae reared on the artificial diets for 3 d (Figure 1B). By feeding larvae with the artificial diets supplemented with fermentation liquids of T30 (clone library of FL-2) and Mc4 (clone library of FL-3), significant changes were observed in the proportion of each species accounting for the bacterial community. Therefore, the bacterial clone libraries of SDT-1, FL-1, FL-2, and FL-3 were further analyzed.

### Statistical Analysis of the Bacterial 16S rRNA Gene Libraries

Four bacterial 16S rRNA gene libraries (SDT-1, FL-1, FL-2, and FL-3) were statistically analyzed **(** **Table 1** **)**. A total of 160 clones of the SDT-1 library were divided into five OTUs, 158 clones of the FL-1 library were divided into five OTUs, 169 clones of the FL-2 library were divided into four OTUs, and 166 clones of the FL-3 library were divided into four OTUs. The coverage of the four clone libraries ranged from 98.7% to 99.4%, indicating that these 16S rRNA gene sequences represented the majority of the bacterial community in larval midgut of the Asian corn borer. The Shannon–Wiener and Simpson indexes indicated that the diversity of bacteria in FL-3 was the lowest among the four clone libraries.

**Table 1:** Characteristics of 16S rRNA gene libraries constructed from midgut bacterial communities in fourth instar larvae of the Asian corn borer.

Library name	Diets	No. ofsequences	No. of OTUSobserved	Good’s coverage	Shannon H	Simpson 1-D
SDT-1	Sterile artificial diets	160	5	99.4%	1.117(1.002–1.204)	0.6287(0.5779–0.6646)
FL-1	Control artificial diets	158	5	98.7%	1.097(0.9829–1.181)	0.6219(0.5669–0.6583)
FL-2	T30-treated artificial diets	169	4	99.4%	1.170(1.065–1.247)	0.6451(0.5953–0.6833)
FL-3	Mc4-treated artificial diets	166	4	99.4%	0.9831(0.8611–1.704)	0.5610(0.4927–0.6131)

**Figure 2:**
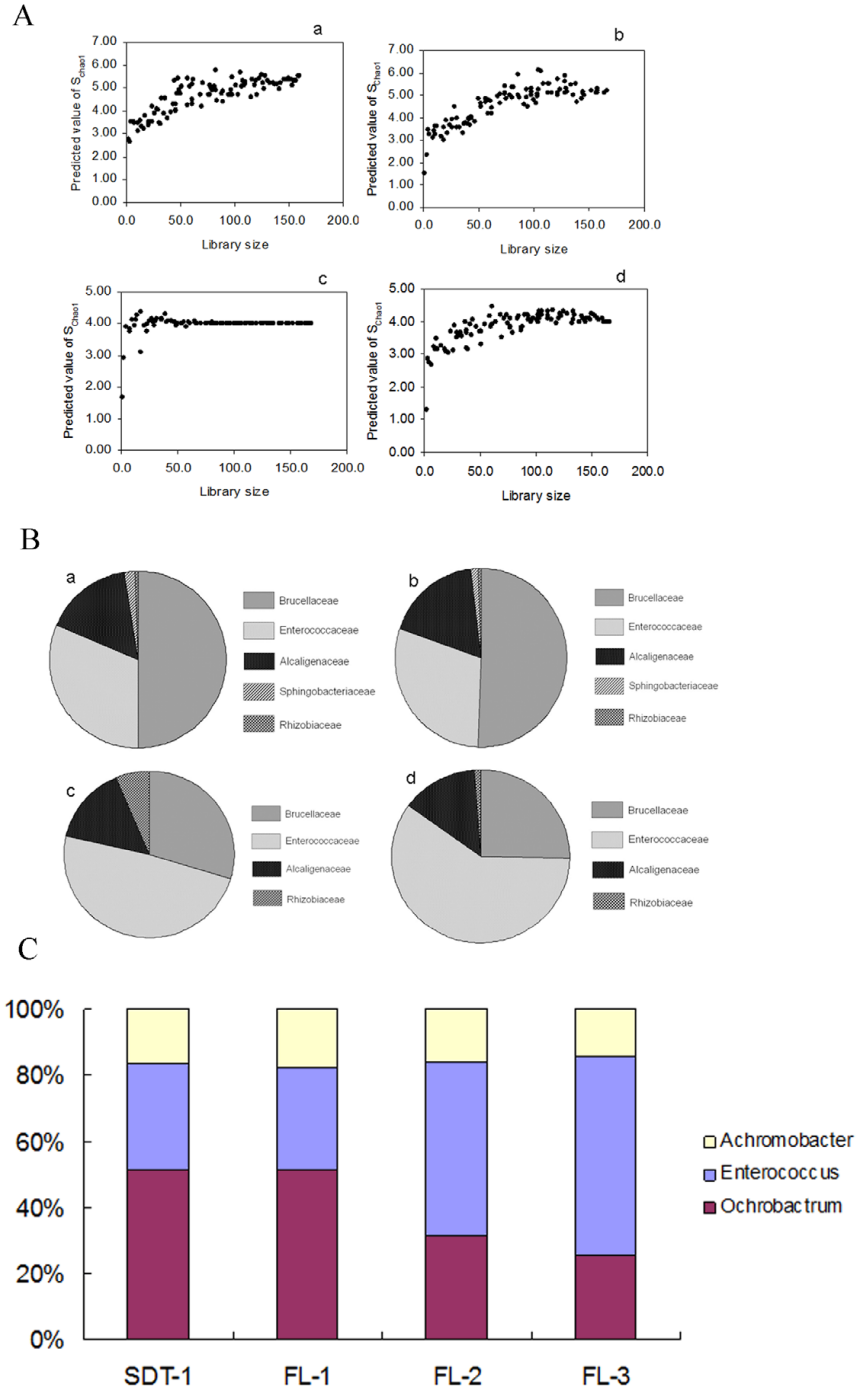
Distribution of midgut bacterial communities between clone libraries of SDT-1, FL-1, FL-2, and FL-3. (**A**) Predicted numbers of phylotypes based on the S_Chao1_ of the 16S rRNA clone libraries. a, clone library of SDT-1; b, clone library of FL-1; c, clone library of FL-2; d, clone library of FL-3. X axis, Bacterial clone numbers in each library; Y axis, Predicted value of S_chao1_. (**B**) The bacterial composition in the larval midgut of fourth instar at the family level. a, clone library of SDT-1; b, clone library of FL-1; c, clone library of FL-2; d, clone library of FL-3. (**C**) Comparison of genera *Achromobacter*, *Enterococcus*, and *Ochrobactrum* distribution in the larval midguts between clone libraries of SDT-1, FL-1, FL-2, and FL-3.

For the four bacterial 16S rRNA gene clone libraries constructed, S_chao1_ was calculated for each library and then plotted to the library size (Figure 2A). The S_chao1_ of the four libraries all reached an asymptote, indicating that the libraries were sufficiently large to yield a stable estimate of the phylotype richness.

### Phylogenetic Analysis of the Midgut Bacterial Communities of the Fourth Instar Larvae

The composition of the bacterial communities was analyzed based on the bacterial 16S rRNA gene sequences obtained from the midgut of the fourth instar larvae reared on sterile artificial diets.

**Figure 3:**
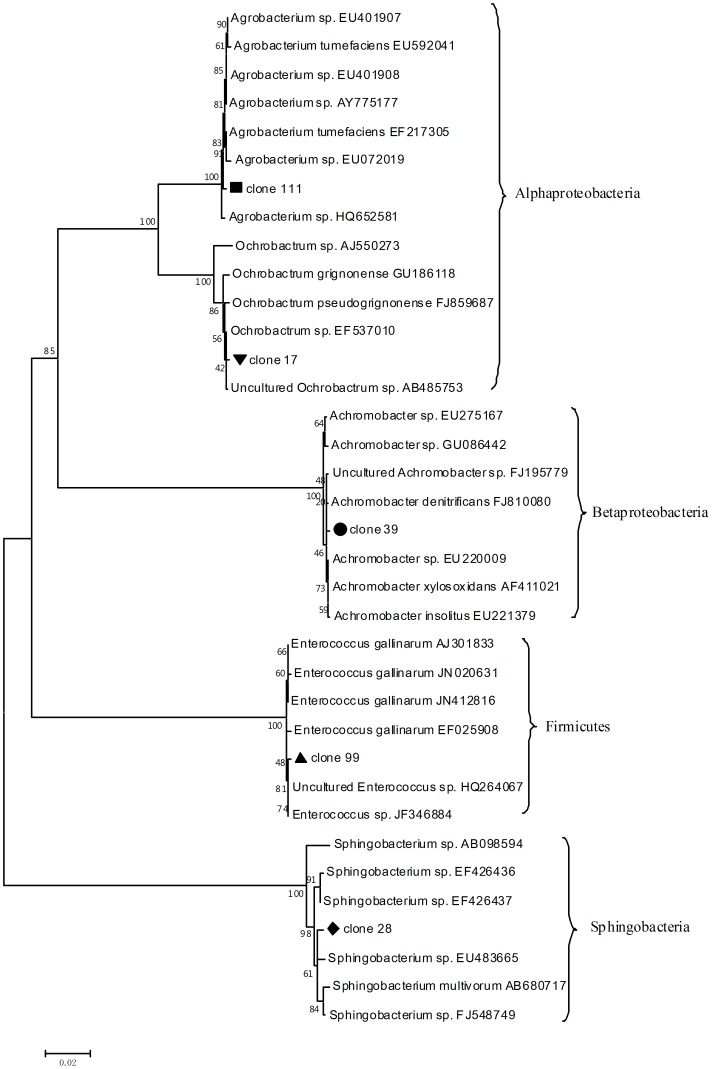
Phylogenetic neighbor-joining trees of representative OTUs in the midgut bacterial clone libraries. The numbers on the branches are bootstrap values obtained from 1000 bootstrap replicates.

At the family level, the dominant families in SDT-1 were Brucellaceae (50.0%), Enterococcaceae (31.2%), and Alcaligenaceae (16.3%) (Figure 2B). The remaining sequences were affiliated with Sphingobacteriaceae (1.9%) and Rhizobiaceae (0.6%) (Figure 2B). All the sequences were clustered into four classes: Alphaproteobacteria (50.6%), Betaproteobacteria (16.3%), Firmicutes (31.2%), and Sphingobacteria (1.9%) (Figure 3).

BLAST searches were performed against the nr-database of the National Center for Biotechnology Information. Table 2 summarizes the BLAST results of the representative OTUs in the clone libraries.

**Table 2:** Partial 16S rDNA sequences isolated in this study.

Closet known relative	Phylogenetically related class	Sequence length (bp)	Identity (%)	Accession no.*
Uncultured *Ochrobactrum* sp.	Alphaproteobacteria	1446	99	AB485753
*Enterococcus* sp. GYPB01	Firmicutes	1524	99	JF346884
*Achromobacter denitrificans*	Betaproteobacteria	1495	99	FJ810080
*Sphingobacterium* sp. GF2B	Sphingobacteria	1494	99	FJ548749
*Agrobacterium* sp. CIP 106281	Alphaproteobacteria	1449	99	EU401908

* The accession number of the closest relative is indicated between brackets.

### Effects of the Transgenic *Trichoderma* Strain Fermentation Liquids on the Larval Midgut Bacterial Communities of the Asian Corn Borer

Among all treatments to the larval midgut, *Ochrobactrum*, *Enterococcus*, and *Achromobacter* were found to be the dominant bacterial microflora. However, significant changes in dominant bacterial communities were observed after feeding on artificial diets supplemented with the fermentation liquids of the transgenic *Trichoderma* strain (Figure 2C).

In SDT-1, *Ochrobactrum* and *Enterococcus* accounted for 50.0% and 31.2% of the 16S rRNA gene sequences, respectively. In FL-1, *Ochrobactrum* and *Enterococcus* accounted for 50.6% and 29.7% of the 16S rRNA gene sequences, respectively. In FL-2, however, these numbers shifted to 29.6% and 49.1%, respectively. A similar trend was observed in FL-3, in which the *Ochrobactrum* and *Enterococcus* members shifted to 25.3% and 59.6% of the 16S rRNA gene sequences, respectively. Additionally, no significant change was detected in the numbers of genus *Achromobacter*. In SDT-1, FL-1, FL-2, and FL-3, *Achromobacter* accounted for 16.3%, 17.7%, 14.8%, and 13.9% of the clones in each library, respectively.

### Bacterial Isolation and Phylogenetic Analysis

*Enterococcus* was isolated from midguts of the larvae reared on the sterile artificial diets. The isolated *Enterococcus* strain could hydrolyze the aesculin content in the medium and then react with an ion to generate a brownish black substance. The colony appeared grayish-white, opaque, round, low-arched, wet, and smooth. The colony exhibited a good growth at 45°C under pH 9.6, and can resist 60°C for 30 min [25].

**Figure 4:**
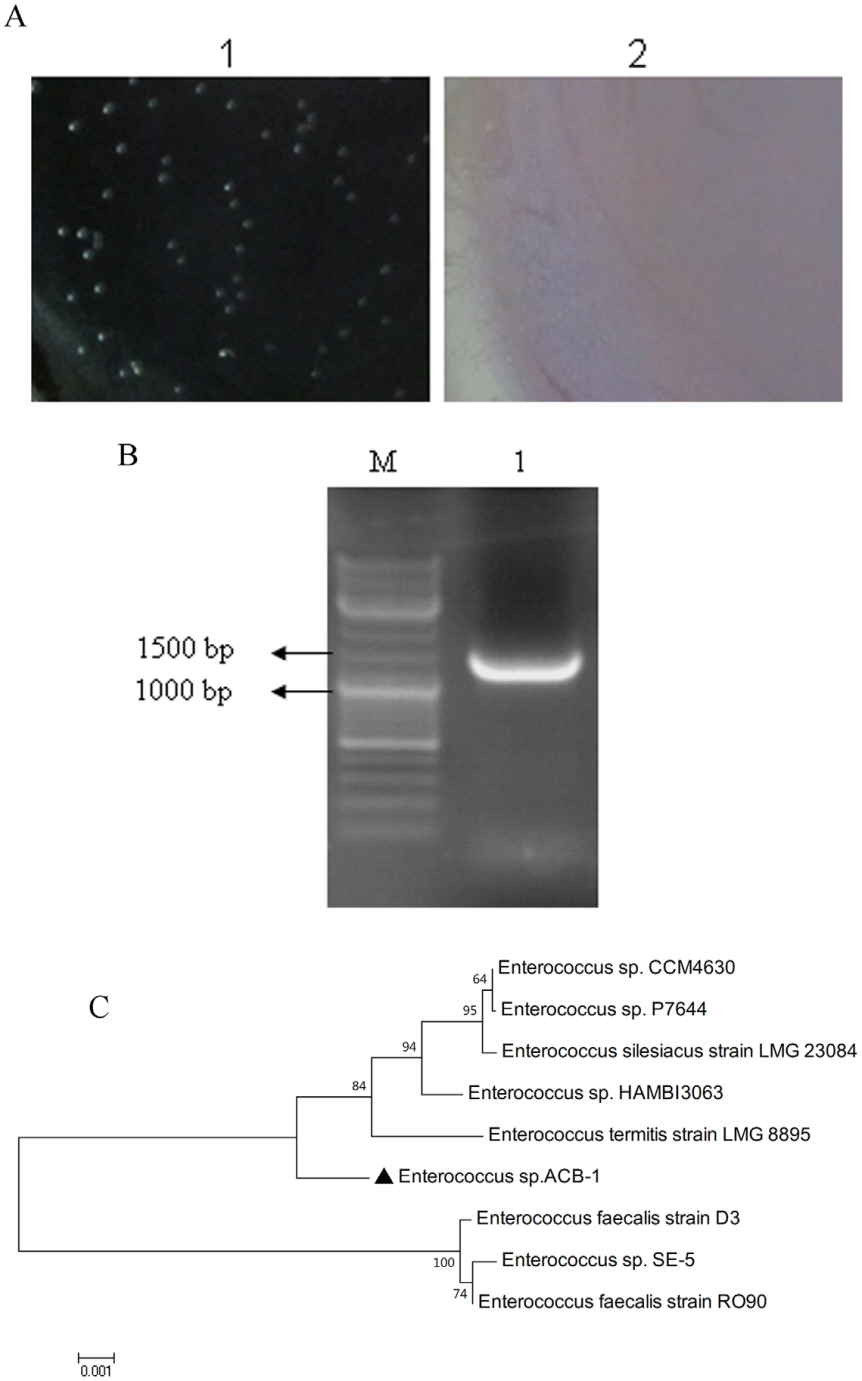
Isolation and characterization of the isolated *Enterococcus* sp. ACB-1 from healthy midgut of the Asian corn borer larvae. (**A**) Colony appearance of the *Enterococcus* sp. ACB-1 growing on *Enterococcus* agar. 1, *Enterococcus* agar with the *Enterococcus* sp. ACB-1 grown on it; 2, *Enterococcus* agar without the *Enterococcus* sp. ACB-1 grown on it. (**B**) 16S rDNA fragment amplification of the *Enterococcus* sp. ACB-1. (**C**) Phylogenetic neighbor-joining trees of the *Enterococcus* sp. ACB-1 (▴). The numbers on the branches are bootstrap values obtained from 1000 bootstrap replicates.

The 16Sr DNA sequence was cloned using the primers 27f and 1492r (Figure 4A). The results of the phylogenetic analysis are indicated in Figure 4B.

### Infection of the Larvae Rearing on the Sterile Artificial Diets

**Figure 5:**
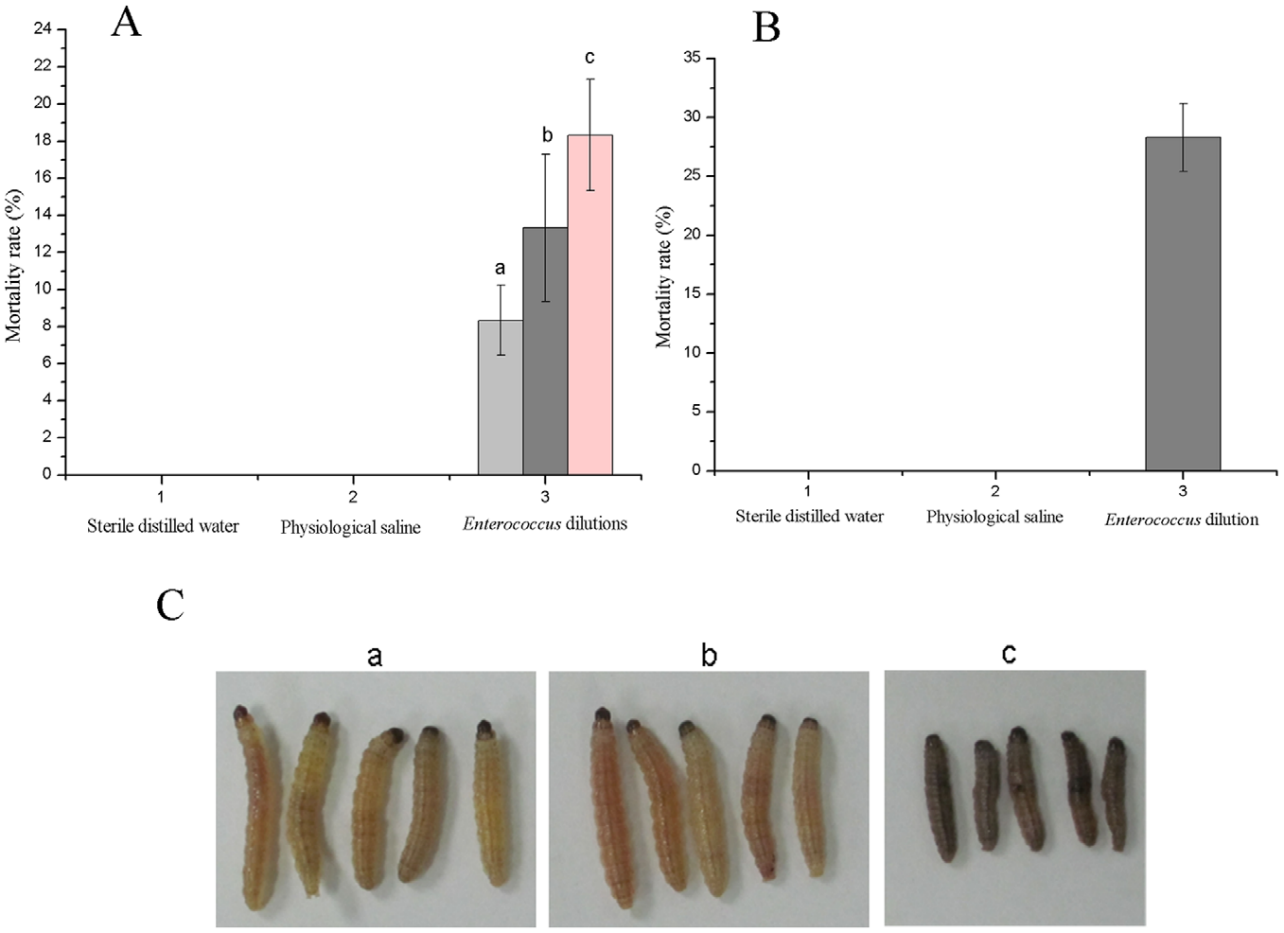
Pathogenic studies of the Asian corn borer larvae by the *Enterococcus* sp. ACB-1. (**A**) Mortality of the Asian corn borer larvae after feeding strain of the *Enterococcus* sp. ACB-1. a, *Enterococcus* sp. ACB-1 suspension with dilution of 10^−3^; b, *Enterococcus* sp. ACB-1 suspension with dilution of 10^−5^; c, *Enterococcus* sp. ACB-1 suspension with dilution of 10^−7^. (**B**) Mortality of the Asian corn borer larvae after injecting strain of the *Enterococcus* sp. ACB-1. (**C**) Larvae appearance after incubation for 5 d by injection strain of the *Enterococcus* sp. ACB-1 with dilution of 10^−5^. a, Sterile distilled water; b, Physiological saline; c, *Enterococcus* sp. ACB-1 suspension with dilution of 10^−5^.

The *Enterococcus* strain was pathogenic for the Asian corn borer larvae. The body of the larvae appeared black after the infection study (Figure 5C). The infectious symptom of the *Enterococcus* strain by injection could be observed after an incubation period of 1 d. After an incubation period of 2 d, the larvae began to die. Mortality reached 28.3% after incubation for 5 d (Figure 5B). The infection study by feeding was analyzed using three dilution degrees of a pure of *Enterococcus* strain culture suspended in physiological saline. A higher *Enterococcus* concentration resulted in a shorter time for the infectious symptom to appear. Larvae infection by injection was more severe than that by feeding. This result was in accordance with the previous report by Lysenko [24]. When using a dilution of 10^−5^ pure *Enterococcus* strain culture by feeding, mortality only reached 13.3% after incubation for 5 d (Figure 5A). No mortality was observed in the physiological saline control and sterile distilled water control in both injection and feeding treatment within 5 d.

## Discussion

Studies on isolating and characterizing the Lepidopteran intestinal bacterial community are limited. The midgut bacterial community of the gypsy moth larvae was reported to be relatively simple [3]. Cabbage white butterfly larvae were shown to contain a simple bacterial community with a species richness ranging from six to 15 [4]. Phylotypes belonging to five genera were identified from the midguts of the laboratory population of the *Helicoverpa armigera* larvae [5]. In this study, we surveyed the changes in the bacterial communities in midgut of fourth instar of the Asian corn borer larvae after feeding on artificial diets supplemented with the supernatant of the fermentation liquids of the transgenic strain to understand the association between the bacterial community imbalance in the midgut and the mortality of the corn borer larvae. Results demonstrated that the observed species richness was five, a number that was relatively simple compared with other gut environments such as in the hindguts of isopteran termites [26]. No report is available on the relationship between the complexity of the bacterial community of an insect and robustness to external factors. However, experimental evidence suggests that species diversity in small grassland plots enhances invasion resistance [27]. A study on the species diversity and invasion resistance in a marine ecosystem also revealed that increased species richness significantly decreased invasion success. That is, a more diverse an ecosystem is more resistant to exotic species [28]. Based on these studies, we infer that the community of the Lepidopteran midgut might be easily damaged and consequently lose its ecological buffering capability.

The intestinal dominant bacterial flora depends on different insect species. *Ochrobactrum*, *Enterococcus*, and *Achromobacter* were identified as the dominant genera, which belonged to the phyla Proteobacteria and Firmicutes, respectively. These results are consistent with the previously reported bacterial communities in other Lepidopteran midguts, such as the *Lymantria dispar*, *H. armigera*, *Bombyx mori*, *Schistocerca gregaria*, [29] and those in the midgut of the natural aphid populations [30]. Although the taxonomic status of the midgut bacterial communities was consistent at the phylum level in the different insects discussed above, significant differences in taxonomic status were observed at the class level. Except for the genus *Enterococcus*, genera *Ochrobactrum* and *Achromobacter* were not detected in other bacterial communities of the Lepidoptera insects’ midguts, such as *Lymantria dispar*, *Pieris rapae*, and *H. armigera*. Similarly, genera *Enterobacter*, *Pantoea*, and *Acinetobacter* detected in the midguts of other Lepidopteran insects were not found in midgut of the Asian corn borer. The bacteria belonging to Sphingobacteria only accounted for less than 2% of the bacterial community in the Asian corn borer midgut, although they were dominant in midgut of the isopteran termites [31], [32], [33]. Proteobacteria has been detected in many insect species, which implies the presence of common Proteobacteria in gut environment of the insect.

We found that the *Trichoderma* fermentation liquids altered the relative proportions of the different midgut flora components. *Ochrobactrum* and *Enterococcus* were detected as the dominant members in all bacterial libraries. In FL-2 and FL-3, the proportion of *Ochrobactrum* was reduced by 50% in the community compared with SDT-1 and FL-1. *Enterococcus*, however, was increased by more than 50%. A variety of factors could facilitate the changes of the microbial community composition. The reason why an improved chitinase activity could facilitate a dynamic change in the midgut microbial community is remains unclear. Chitin, a component of the exoskeleton and gut lining of insects, is a metabolic target for pest control agents. Both the chitinases produced by *Trichoderma* and *Metarhizium* could degrade chitin. Ingested chitinase can damage the peritrophic membrane, potentially facilitating the changes in intestinal bacterial community structure. In addition, chitin degradation in the midgut and peritrophic membrane might result in the midgut cell electrolyte leakage, possibly providing a new source of nutrition for the midgut bacterial community. When the larvae were treated with chitinase fermentation liquids, the physiological activity of the insect was affected because of chitin content degradation on the peritrophic membrane structure of the larvae. Moreover, intestinal microorganisms were also closely related to the physiological activity of the insect. Based on our results, the *Enterococcus* community increased when the larvae were fed with artificial diets supplemented with the chitinase fermentation liquids. The typical ecological niche for the *Enterococcus* species is the intestine of humans and other animals. Enterococci, which were firmly established as major nosocomial pathogens, have also been related to human diseases. A large number of enterococci were also found in the intestinal tract of silkworms, the presence of which is not harmful under normal conditions. However, silkworms could be infectious when the silkworm the larvae are suffering from poor physiological activity. *Enterococcus mundtii* was shown to be associated with the flacherie disease of the silkworm larvae reared on artificial diets. Silkworm larvae infection analysis with *E. mundtii* was conducted by using pure *E. mundtii* culture in a nutrient broth sprinkled directly on the artificial diet. The larvae were reared under germfree conditions. The results indicated that the germfree larvae displayed typical bacterial infection symptoms after a few days. The number of days varied according to the quality and concentration of the *E. mundtii* inoculum. The infection could be achieved at low *E. mundtii* concentrations added to the diet. The infection could also enter the body cavity after multiplying in the larval gut [34]. Lysenko used two kinds of methods to prove the pathogenicity of the *Streptocossus* strains on healthy silkworm larvae through feeding and injection [24]. Silkworm larvae mortality reached 100% after injecting the *Streptocossus* strains into the larval cavity. In our study, we conducted the infection study of the Asian corn borer larvae using *Enterococcus* sp. isolated from midgut of the healthy the Asian corn borer larvae. Results indicated that an increase of the *Enterococcus* community in the larval midgut could indeed increase the mortality of the larvae. We remain unaware of why injecting the *Enterococcus* strain into the body cavity of the Asian corn borer larvae could result in larval mortality. We speculated that the *Enterococcus* strain could enter into the larval midgut after multiplying in the body cavity. Based on the results, the increased *Enterococcus* community in the larval midgut could be an important factor resulting in the death of the Asian corn borer larvae.

Bacterial diversity changes caused by gastro-intestinal diseases have been reported in humans. *Bacteroides* spp., *Clostridium coccoides*, and *Clostridium leptum* are considered to be the dominant populations in the human fecal microflora [35], [36]. The results indicated that patients who suffered Crohn’s disease had a reduced diversity of the bacterial phylum Firmicutes [37]. Furthermore, the numbers of lactobacilli, *Clostridium coccoides*, and *Clostridium leptum* decreased in fecal microflora, whereas the number of enterobacteria increased when the patients were suffering inflammatory bowel diseases. The white plague disease (WPD) is a prevalent coral disease that mainly affects massive coral species. Shifts in bacterial communities were observed when the coral species were infected with WPD. A significant increase in Alphaproteobacteria was also observed in WPD-affected coral species. A concomitant decrease in the Beta- and Gammaproteobacteria was observed [38]. The number of *Enterococcus* clones increased, followed by a reduction of the *Ochrobactrum* clones. *Enterococcus* species were present in the larvae of silkworm [39] and gypsy moth [3] regardless of diets. These results were in accordance with our findings, in which *Enterococcus* species were present in all the larvae fed with different artificial diets. Other dominant bacteria types such as the *Enterobacter* species in lepidopteran midguts were not detected in this study.

Exploring the association between the Asian corn borer larvae midgut microbial community imbalance and larval mortality will enrich our understanding of the mechanism induced by chitinase from the transgenic *Trichoderma* strain against corn borers. Further analysis of this midgut ecosystem, including the dynamic changes in the midgut microbial composition using the metagenomic method, will provide more useful markers for the biocontrol of insect pests.
